# From map to blueprint: the plant pan-genome unraveling genetic mysteries and powering precision breeding

**DOI:** 10.3389/fpls.2025.1673637

**Published:** 2025-09-23

**Authors:** Chong Liu, Hang Xu, Zheng Li, Yukun Wang, Jiaxian Zhang, Siwei Qiao, Hao Zhang

**Affiliations:** Institute of Special Animal and Plant Sciences, Chinese Academy of Agricultural Sciences, Changchun, China

**Keywords:** plant pan-genome, genome assembly, sequencing technology, plant breeding, genetic variation

## Abstract

With the rapid advancement of sequencing technologies and bioinformatics, coupled with significant progress in sequencing efficiency and reduced costs, substantial breakthroughs have been achieved in plant functional genomics, evolutionary genetics, and molecular breeding. However, as research deepens, accumulating evidence demonstrates that reference genomes derived from a single individual fail to adequately represent the genetic diversity of entire species. This limitation has catalyzed the emergence of the pan-genome concept. Pan-genome research now stands at the forefront of plant genomics, serving as a pivotal area of focus. Its application in plant studies has unveiled extensive genetic variations, identified numerous novel genes, and significantly enhanced our understanding of genetic diversity within relevant species. This review comprehensively summarizes recent progress in plant pan-genome research, construction methodologies, current applications in plant science, and key achievements. Finally, we outline future research directions, aiming to provide a reference for theoretical and applied pan-genome studies while offering novel perspectives for deciphering the genetic basis of plant breeding, evolutionary domestication, and phenotypic diversity.

## 1 Introduction

### 1.1 Overview of plant pan-genome development

The pan-genome represents the complete set of genes present within a species, encompassing both the core genome—shared by all individuals—and the accessory or variable genome—present only in some individuals, often contributing to phenotypic diversity and adaptation. The term “pan-genome” originates from the Greek word παν (pan), meaning “all,” reflecting its aim to capture the entirety of genetic repertoire across a species. This concept was first introduced by Tettelin et al (Tettelin et al., 2005). in 2005 during genomic studies of Streptococcus agalactiae, where it was observed that a single reference genome could not represent the full gene content of a bacterial species due to extensive horizontal gene transfer and presence–absence variations (PAV) (Tettelin et al., 2005). In 2007, Morgante et al. (2007) extended the pan-genome concept to plants, highlighting its potential to address the limitations of single-reference genomes in capturing the genetic diversity of higher eukaryotes. However, progress in plant pan-genomics was initially slow, hampered by high sequencing costs, technological constraints, and the lower prevalence of large-scale PAV in plants compared to microbes.

**Figure 1:**
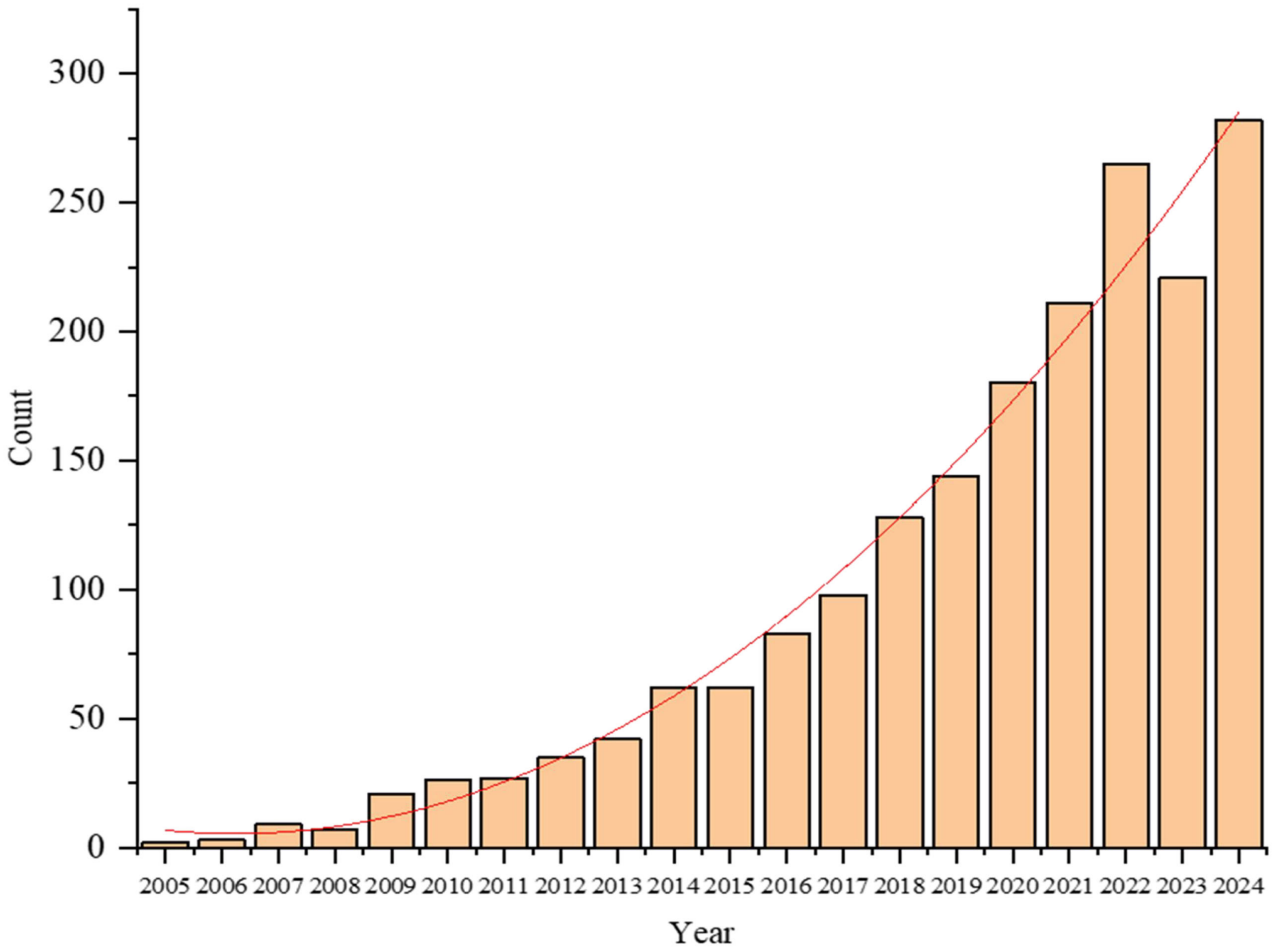
Articles related to pan-genome found in PubMed.

A turning point came in 2014, when several landmark studies demonstrated the feasibility and value of pan-genome approaches in plants. Li et al. (2014) constructed the first plant pan-genome for wild soybean (*Glycine soja*), revealing extensive structural variations lost during domestication. In the same year, Schatz et al. (2014) published a pan-genome for cultivated rice (*Oryza sativa*), and Hirsch et al. (2014) developed a pan-transcriptome for maize (*Zea mays*). These studies collectively marked the emergence of plant pan-genomics as a distinct and impactful research field (Jayakodi et al., 2021), the related reports on pan-genomes have shown a significant growth trend, as shown in **Figure 1**. Since then, pan-genome research has rapidly expanded, driven by advances in sequencing technologies and bioinformatics. Pan-genomes are now recognized as essential resources for capturing species-wide genetic diversity, identifying novel genes, and linking structural variations (SVs) to agronomic traits (Chaudhari et al., 2016). The core genome consists of evolutionarily conserved genes characterized by low Single Nucleotide Polymorphisms (SNPs) density and extended sequence length (Tao et al., 2019a; 2019b), typically governing fundamental cellular processes (Jayakodi et al., 2021). Studies emphasize the critical role of plant pan-genome in non-coding RNA discovery, crop improvement (Tahir Ul Qamar et al., 2020), they have been constructed for a growing number of plant species, as shown in **Table 1**, enabling deeper insights into evolutionary history, domestication processes, and functional genomics.

**Table 1:** Representative achievements of plant pan-genome research progress.

Species	Year	Construction strategy	Number of individuals	Pan-genome size (Mb)	Key findings	References
*Glycine soja Siebold& Zucc.*	2014	*De novo*	7	NA	The first construction of the plant pan-genome; Reveal the loss of a large number of SVs during domestication.	(Li et al., 2014)
*Zea mays L.*	2014	Pan-transcriptome	503	NA	Discover non-reference gene regulation of stress resistance; Propose the framework of the corn pan-genome.	(Hirsch et al., 2014)
*Brassica oleracea L.*	2016	Iterative assembly	10	587	Several PAV genes were annotated for disease resistance, flowering time, glucosinolates metabolism and vitamin biosynthesis.	(Golicz et al., 2016)
*Oryza sativa L.*	2018	*De novo*	3010	638	Build high-quality pan-genome; Support the hypothesis of independent domestication of indica-japonica rice.	(Wang et al., 2018)
*Solanum Lycopersicum L.*	2019	*De novo*	725	1300	4,873 reference genome deletion genes were discovered; Identify the rare allele TomLoxC that regulates flavor.	(Gao et al., 2019)
*Helianthus annuus L.*	2019	Map-to-Pan	493	NA	10% of the cultivated species’ genes are derived from wild species and contain disease-resistant genes.	(Hubner et al., 2019)
*Glucine max(L.)*	2021	Map-to-Pan	204	108	Discovered 108 Mb of novel non-reference sequences, including 3,621 protein-coding genes that do not exist in the reference genome of soybean “Williams 82”.	(Torkamaneh et al., 2021)
*Arachis hypogaea*	2025	Graph-Based	269	NA	Structural variations in the AhARF2–2 gene regulate grain size. The AhCKX6 gene is significantly associated with grain weight.	(Zhao et al., 2025)
*Solanum*	2025	*De novo*	22	NA	Created a single fused CLV3 allele that modulates fruit organ number alongside an enzymatic gene controlling the same trait.	(Benoit et al., 2025)

Plant pan-genome construction involves integrating genomic sequences from multiple accessions or varieties, facilitating the identification of core genes—which are evolutionarily conserved and often involved in essential biological processes—and accessory genes, which may confer adaptive traits. Beyond gene content, pan-genomic analyses now also encompass regulatory variations and non-coding sequences, providing a more holistic view of genomic architecture (Gordon et al., 2017). The adoption of pan-genomes has significantly advanced studies in non-coding RNA discovery, crop improvement, and association genetics (Li et al., 2014), enabling researchers to connect genetic diversity with phenotypic outcomes more accurately than ever before (Shang et al., 2022).

### 1.2 Advantages and trends of pan-genome research

pan-genome offer significant research advantages: enhancing data breadth by capturing species-wide genomic diversity to overcome limitations of single-reference genomes (Zhao et al., 2025); improving analytical precision through genome-scale structural variation detection for advanced breeding and domestication studies (Chen et al., 2023); deepening investigative focus on unique genes to elucidate mechanisms underlying accession-specific traits (Jiao et al., 2025; Yang et al., 2025); elevating multi-omics integration by combining pan-genomics with population resequencing, transcriptomics, and metabolomics (Li et al., 2025a). Pan-genomics provides a comprehensive approach to accessing complete gene catalogs, mitigating reference biases when studying divergent lineages (He et al., 2023). Its exploration within plant genomics continues to expand, demonstrating significant potential for genomic research. pan-genome further aid taxonomic classification, precise determination of population-level gene content, and understanding of organismal adaptive strategies (Olanrewaju et al., 2021; Zhang et al., 2025a).

## 2 Construction of plant pan-genome

### 2.1 Sample selection

Selecting appropriate materials for high-quality genome assembly constitutes a prerequisite for pan-genome studies (Feng et al., 2025). Two critical factors—sample size and genetic diversity—directly determine pan-genome size, core/accessory gene proportions, and overall quality. Incorporating both wild and modern cultivated accessions enriches genetic variation and comprehensively reveals dynamic genomic changes during domestication. For example, Wang et al. constructed a high-quality Asian rice pan-genome using 3,010 accessions from 89 countries, representing >95% of genetic diversity from 780,000 global rice germplasms (Wang et al., 2018).The selection of materials needs to be representative enough, the geographical location should be as wide as possible, and the materials can cover the whole country or even the world; second, there should be enough differences between the materials, and the phenotype should be comprehensive and abundant; Third, the types of materials should be complete, and the proportion of wild materials and cultivated materials should be appropriately adjusted (Zhao et al., 2018; Guo et al., 2025). Therefore, the addition of relevant wild relatives to the pan-genome study of crops can efficiently uncover the beneficial genes lost during crop domestication. Constructing a phylogenetic tree of samples based on the identified SNPs can facilitate the selection process (She et al., 2025).

### 2.2 Construction strategies

For well-annotated genomes, analyzing per-gene PAV identifies core and accessory genes, yielding a gene-based pan-genome (Danilevicz et al., 2020). While conceptually straightforward and widely used in prokaryotes, this approach ignores sequence-level variations beyond genes, inadequately capturing genomic diversity (Michael and VanBuren, 2020). Each assembly method has its own characteristics and application scenarios (**Table 2**). Thus, complex eukaryotes typically adopt sequence-based pan-genome, classified into four strategies, the four primary strategies for constructing plant pan-genomes each possess distinct characteristics, advantages, and limitations, making them suitable for different research scenarios. The choice among them should be guided by several key factors: firstly, consider the availability and quality of the reference genome; secondly, the number of individuals to be sequenced and their genetic diversity; thirdly, the research objective, specifically whether it focuses on the PAV, SV, or both; finally, the available computing resources and sequencing budget.

**Table 2:** Comparison of construction methods for plant pan-genome.

Methods	Core principle	Advantage	Disadvantage	Applicable scene
Iterative assembly	Based on the reference genome, gradually add unaligned sequences.	Low computing needs; Cost control; Can be incremental updating genome.	Dependent on the initial reference quality; Difficult to detect complex SVs.	For high quality reference genome; Medium-sized groups.
*De novo* assembly	Each individual was independently sequenced and *de novo* assembled, then integrated through multi-genome alignment	Do not rely on the reference genome; Can detect overall variation; Applicable to species without reference.	High consumption of computing resources; High sequencing cost; Poor applicability to large genomes/large populations.	Species research without a reference genome; High precision SV mining.
Map-to-Pan	Compare all individuals with the reference genome, merge non-reference sequences to remove redundancy, and then combine them with the reference.	Process simple efficient; Avoid iterative sequence deviation; Suitable for large scale sample.	Still relying on the reference genome; May miss regions that differ significantly from the reference genome.	Very large scale group; Resources economical analysis.
Graph-based assembly	Represent sequences and joins with a graph structure and integrate all variations	Eliminate reference deviation; Multi-scale mutation; Support efficient genotype identification.	Algorithm is complex to build; Analysis tools are not yet mature; Large demand for data storage.	The mainstream direction of high species diversity accurate breeding application in the future.

#### 2.2.1 Iterative assembly

**Figure 2:**
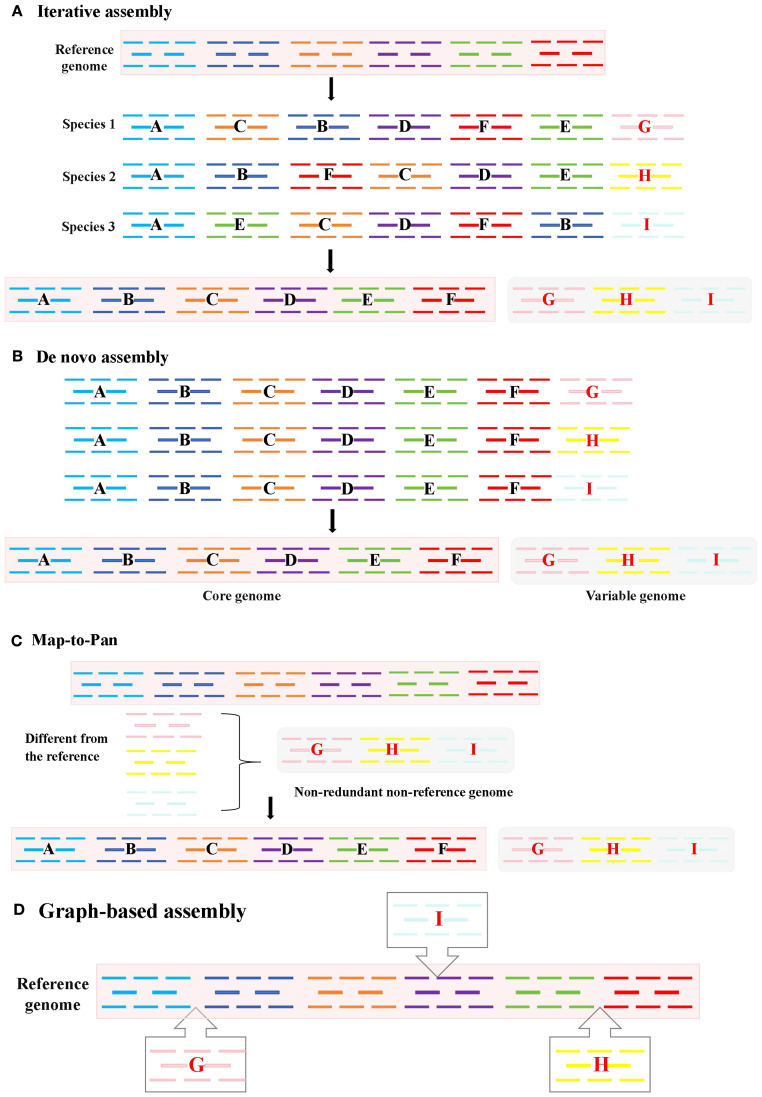
Four methods for constructing pan-genomes: **(A)** Iterative assembly. After comparing the entire genome, fragments that did not align to the reference genome are added to the reference genome; **(B)** *De novo* assembly. From the assembly of multiple individuals’ genomes, the core and non-core genomes are identified; **(C)** Map-to-Pan. The genomes of all individuals are compared with the reference genome separately, redundant parts are removed to obtain non-redundant non-reference genomes, and then the reference genomes are merged; **(D)** Graph-based assembly. Variants are marked in a graphical form.

Iterative Assembly is most appropriate for projects with a high-quality reference genome and a moderate number of samples (tens to a few hundred). Its cost-effectiveness and computational efficiency make it ideal for incrementally expanding the gene repertoire of a species where detecting complex SVs is not the primary goal (Golicz et al., 2016; Jiao and Schneeberger, 2020).This reference-guided method iteratively aligns individual genomes to a reference, identifies non-reference sequences, and expands the reference through sequential integration (**Figure 2A**).Specifically, through iterative comparison and assembling unaligned reads, it can be summarized into the following three steps. First, the short reads obtained in high throughput were aligned to the species reference genome; Then, the reads that were not aligned to the reference genome were extracted and assembled. Finally, the newly assembled Contigs were integrated into the reference genome sequence to construct a pan-genome of the species. This method has low sequencing cost and computational resource requirements, and can comprehensively resolve intra-species variation, so it is widely used in pan-genome studies of many plants, including pan-genome studies of *Arabidopsis* (Jiao and Schneeberger, 2020), *Brassica oleracea* (Golicz et al., 2016), *Cajanus cajan* (Zhao et al., 2020), etc.

#### 2.2.2 *De novo* assembly

*De novo* Assembly is the preferred choice when no reference genome exists or when the aim is to achieve the most comprehensive detection of SVs, including those in complex and repetitive regions. This method is mandatory for constructing pan-genomes of non-model organisms. However, it requires substantial computational power and high-depth sequencing data, making it less feasible for large populations (e.g., >100 individuals) or species with very large genomes (Wang et al., 2018; Tao et al., 2019a; 2019b). A method for constructing a pan-genome via *de novo* genome assembly involves performing *de novo* assembly and annotation for multiple individuals separately (**Figure 2B**). The SOAP *de novo* software is frequently used for assembly, making this a widely applied approach. The strategy of using *de novo* assembly to construct a species’ pan-genome entails sequencing and assembling each sample individually to obtain its complete genome sequence; these sequences are then merged after redundancy removal to form a pan-genome encompassing all individual genome sequences of the species. *De novo* assembly of individual genomes can be accomplished without relying on a reference genome. It is divided into two strategies: short-read assembly and long-read assembly. This method offers the advantage of detecting more SVs but demands substantial computing resources and high sample sequencing depth, rendering it unsuitable for analyzing species with large genomes or large-scale populations. Short-read assembly can also capture extensive genetic information of a species without reference to a genome (Pinosio et al., 2016; Tao et al., 2019a; 2019b). For instance, researchers constructed pan-genome for Glycine max and Medicago sativa using 7 wild soybean accessions and 15 alfalfa accessions, respectively. Through *de novo* assembly of these varieties’ genomes, they identified significant genetic information absent from reference genomes. Long-read assembly has become increasingly prevalent in species pan-genome construction in recent years (Jiao and Schneeberger, 2017; Zhao et al., 2018). Notably, assemblies generated from long reads far surpass those from short reads in terms of sequence continuity and completeness, laying a crucial foundation for acquiring comprehensive variation information of species. *De novo* assembly-based pan-genome construction has also been widely applied in research on the pan-genome of *Glycine max*, *Brassica napus*, *Solanum lycopersicum*, and *Oryza sativa* (Zhao et al., 2018).

#### 2.2.3 Map-to-pan

Map-to-Pan offers a robust balance between completeness and efficiency for studies involving a large number of individuals (hundreds to thousands). It is particularly advantageous when working with a well-established reference genome and aims to efficiently catalog non-reference sequences while avoiding the potential biases introduced by the order of sample processing in iterative assembly. It is a resource-economic strategy for large-scale population studies (Torkamaneh et al., 2021). This method aligns all individuals to the reference simultaneously. Non-reference sequences are merged, deduplicated, and combined with the reference (**Figure 2C**). Applied in sunflower using 287 cultivars, 17 landraces, and 189 wild accessions, it revealed 10% of cultivated genes originated from wild relatives—primarily disease-resistance genes. Importantly, this method features a simple workflow and avoids biases from varying iterative orders in iterative assembly, making it widely used in recent studies—for example, in pan-genome construction for *Oryza sativa* (Wang et al., 2018), *Solanum lycopersicum* (Gao et al., 2019), *Malus domestica* (Sun et al., 2020), *Glycine max* (Torkamaneh et al., 2021), and *Gossypium hirsutum* (Li et al., 2021).

#### 2.2.4 Graph-based assembly

Graph-based assembly is at the cutting-edge level and is rapidly becoming the standard method for future pan-genome research, as it minimizes the deviation of the reference genome to the greatest extent (Garrison et al., 2018). However, building this technology requires a significant amount of computing resources, thus increasing the cost significantly. It is suitable for complete genome maps as it provides a more superior foundation for showcasing species diversity (Bayer et al., 2020). Advancements in third-generation sequencing technologies, such as those using PacBio and Oxford Nanopore platforms, generate significantly longer reads (**Figure 2D**). When combined with sophisticated assembly algorithms, these technologies enable the construction of large, complex plant genomes at unprecedented resolution (Koren et al., 2017; Cheng et al., 2021), improving sequencing accuracy and overcoming the read-length limitations of second-generation sequencing (Niu et al., 2022). This facilitates the resolution of biological questions previously intractable with low-quality reference genomes (Marcus et al., 2014). For example, the 25.4 Gb high-quality genome of *Pinus tabuliformis* —the largest gymnosperm genome published to date—was assembled using long-read PacBio and Hi-C sequencing (Niu et al., 2022). Leveraging relationships between reference and variant sequences, graph-based pan-genome represent sequences as nodes and connections as edges. Tools like the Variation Graph Toolkit (Garrison et al., 2018), Minigraph-Cactus (Li et al., 2020), Seven Bridges GRAF Pipeline (Patron et al., 2019), and PanGenie (Ebler et al., 2022) have been developed for graph-based pan-genome construction. Methods based on variant graph assembly adopt *de novo* strategies, generating graphs through fine-grained comparisons of high-quality genomes to capture all forms of genetic variation, comprehensively representing genomic diversity. Graph-based pan-genome have been constructed for *Glycine max* (Liu et al., 2020), *Oryza sativa* (Qin et al., 2021), *Sorghum bicolor* (Tao et al., 2021), *Cucumis sativus* (Li et al., 2022), *Pennisetum glaucum* (Yan et al., 2023), and others, representing the current and future mainstream of plant pangenomics. Stored in graph format, these pan-genome link reference genomes with genetic variations, encompassing all multi-scale variants and the entire genetic repertoire of a species (Bayer et al., 2020). By using graphs to represent diversity and variation relative to a reference, graph-based assembly strategies advance plant pangenomics (Tao et al., 2019a), as demonstrated in *Cucumis sativus* (Li et al., 2022) and *Cucumis melo* (Vaughn et al., 2022). The transition from linear to graph-based pan-genome is particularly impactful for crop breeding, as crop improvement relies heavily on associating genetic variants with agronomic traits. The precise variant information identified by graph-based pan-genome theoretically enhances the accuracy of quantitative trait locus (QTL) mapping and genome-wide association studies (GWAS) (Tao et al., 2019a). These developments provide valuable insights into plant domestication and breeding histories.

### 2.3 Practical considerations in construction

#### 2.3.1 Minimum cohort size

As the required size depends on the genetic diversity of the species. The goal is to reach a saturation point where adding new accessions yields diminishing returns in discovering non-core genes. This is typically assessed by plotting the number of newly discovered genes against the number of sequenced individuals. For highly diverse species like maize or barley, hundreds of accessions may be needed, while for a genetically narrow crop, dozens might suffice (Jayakodi et al., 2021).

#### 2.3.2 Coverage targets

For short-read *de novo* assembly, a sequencing depth of >50x is generally recommended to ensure assembly continuity and accuracy. For long-read assembly (PacBio HiFi, ONT), >20x coverage can often produce high-quality contigs, but higher depth (30-50x) improves consensus accuracy and haplotype resolution (Cochetel et al., 2023).

#### 2.3.3 Quality control benchmarks

The quality of individual assemblies is paramount. Key metrics must be reported for each accession and the final pan-genome (Sun et al., 2020). Firstly, contiguity: N50 (the contig/scaffold length at which 50% of the genome is assembled) should be as high as possible, ideally in the multi-megabase range for scaffold N50 with Hi-C data; Secondly, completeness: BUSCO (Benchmarking Universal Single-Copy Orthologs) scores are essential. A high-quality plant genome assembly should have >95% complete BUSCOs (most of which should be single-copy), indicating comprehensive gene space representation. Finally, accuracy: QV (Quality Value) estimates the base-level accuracy of the assembly. A QV of 40 corresponds to a 99.99% base accuracy, which is a benchmark for a high-quality, error-corrected long-read assembly (e.g., PacBio HiFi).

### 2.4 Benchmarking in construction

#### 2.4.1 Recall and precision

Different construction methods exhibit trade-offs. *De novo* assembly-based methods generally have high recall (they find more true SVs) but can suffer from lower precision (more false positives due to assembly errors in repetitive regions). Map-to-Pan methods often have higher precision but lower recall, as they may miss SVs that are too divergent from the reference to map. Graph-based methods aim to balance both but require sophisticated genotyping tools (e.g., PanGenie, vg) to achieve high accuracy (Ebler et al., 2022).

#### 2.4.2 PAV consistency

PAV calls can vary significantly between methods. A gene might be called “absent” in a Map-to-Pan approach if reads cannot be confidently mapped, but *de novo* assembly of the same data might reveal a highly divergent functional homolog. This lack of consistency is a major challenge, and studies should validate key PAVs experimentally.

#### 2.4.3 Impact on downstream analyses

The choice of variant set profoundly affects genetic analyses. Incorporating SVs and PAVs alongside SNPs consistently leads to: Novel Significant Loci: GWAS and QTL mapping identify large-effect associations that are completely missed by SNP-only analyses (Alonge et al., 2020). Increased Explained Variance: Causal SVs/PAVs often account for a larger proportion of the phenotypic variance, leading to more robust biological models.

## 3 Applications and major achievements of pan-genomics in plant research

Recent advances in next-generation sequencing technologies have propelled pan-genomics to the forefront of plant research (Golicz et al., 2016), catalyzing substantial progress in this field. Decoding genomic information has enabled critical examination of genomic architectures, evolutionary origins, domestication patterns, and artificial selection dynamics across diverse plant species (Pinosio et al., 2016). To address these questions, scientists employ integrated approaches spanning genomics, epigenomics, molecular cytogenetics, and population genetics. Investigating plant genomic content provides critical guidance for identifying elite germplasm resources and developing novel cultivars for crops and medicinal plants. pan-genome surpass single-reference genomes by comprehensively representing population-level genomic variation while facilitating enhanced variant discovery and utilization (Zhao et al., 2025). Integrating pan-genomics with cutting-edge genomic technologies enables efficient and precise identification of genetic variations in germplasm resources, establishing pan-genome research as a pivotal domain in contemporary plant genomics (Jiao et al., 2025).

### 3.1 Applications of pan-genome in mining novel genes

Gene mining based on pan-genome can fully utilize multi-scale genetic variations to improve the accuracy of gene mapping. Integrating pan-genome with transcriptomes, metabolomes and other omics data, and employing strategies such as QTL mapping and genome-wide association study, GWAS, enables rapid identification of genes associated with the formation of important traits. Alonge et al. (2020) identified 238,490 structural variations through pangenomic analysis of 100 tomato lines; RNA sequencing showed that gene expression is extensively affected by these structural variations, with some being associated with important agronomic traits including fruit size, weight and flavor quality. Li et al. (2021) identified 162 QTLs related to fiber quality, yield and flowering time through analysis of the cotton pan-genome, 84 of which were newly discovered through pangenomic research. Pan-genome serve as a powerful tool for in-depth exploration of the associations between agronomic traits and genomic variations, particularly chromosomal structural variations. Niu et al. (2024) constructed 127,421 gene models from 86 accessions, identified a hub gene *BcaFUL.B7* using a pangenomic platform, and characterized pod shatter resistance (PSR) through QTL-seq and co-expression analysis. A comprehensive pan-genome constructed using 20 wild and cultivated barley varieties revealed that PAV genes are frequently associated with resistance gene homologs, which may have profound implications for the utilization of barley germplasm resources, understanding the molecular mechanisms underlying important agronomic traits, and breeding elite varieties with high quality, high yield and stress tolerance (Jayakodi et al., 2021). Before implementing genomic selection, the only way to assess the full range of genetic diversity within a species is by constructing a pan-genome, thereby allowing the use of a broader set of species-specific markers during model training.

### 3.2 Applications of pan-genome in plant breeding

Pan-genome research is bridging the gap from “gene discovery” to “breeding application”. For instance, the tomato pan-genome uncovered a rare allele of *TomLoxC* contributing to flavor intensity; molecular markers developed from this discovery are now utilized in marker-assisted selection to enhance fruit quality in breeding lines (Gao et al., 2019). Similarly, the extensive catalog of structural variations from the rice super pan-genome provides an invaluable resource for deploying broad-spectrum disease resistance genes through targeted gene pyramiding, a strategy crucial for developing resilient cultivars (Wang et al., 2018; Shang et al., 2022). Pan-genomes facilitate the recovery of beneficial alleles lost during domestication, as demonstrated in soybean, where wild relative genes are being reintroduced into elite backgrounds to improve traits like protein content and stress tolerance, it provides a solution for breaking through the genetic bottleneck of cultivated species (Liu et al., 2020). Yu et al. (2024) have for the first time systematically revealed the genetic basis of the variation in Ganoderma lucidum spore production, and clarified the crucial role of the *MSH4* gene in spore formation. This plays a significant role in the breeding of Ganoderma lucidum, helping to increase the yield of spore powder. By encompassing a species’ full genetic diversity, pan-genomes provide the complete variant set necessary for building superior genomic selection models, significantly accelerating the improvement of complex polygenic traits such as yield and adaptation (Bayer et al., 2020). Finally, the massive genotype and variation information generated by the pan-genome serves as the foundation for the efficient implementation of modern breeding technologies such as genomic selection and gene editing.

### 3.3 Applications of pan-genome in plant evolution and domestication

Plant pan-genome exhibit high plasticity and contain richer genetic diversity within species, thus showing great potential in population evolution analysis, tracing species origin and evolution, and plant domestication improvement. Since domestication and breeding primarily utilize the variable components within a species, for any species, non-core genes precisely provide important sources of variation for domestication and breeding, and are often closely associated with key agronomic trait of varieties (Li et al., 2014). Although non-core genes do not directly control basic metabolic processes during species growth, the traits they regulate help enhance the adaptability of the genome to the environment (Bayer et al., 2020; Castillo-Ramirez, 2022). Conducting crop pan-genome research to explore the impact of non-core genes on important crop traits has become a major trend in genomic research. Pangenomic analysis can identify potential stress-responsive genes in individuals with untapped genomic diversity among wild relatives of crops (Tang et al., 2022). Information obtained from these pangenomic analyses can be applied to breed climate resilience in existing crops or re-domesticate crops by incorporating environmental adaptation traits (Petereit et al., 2022). Zhang et al.(Zhang et al., 2025b) discovered that the formation of hybrid species can involve both genetic substitution of parental genes and the development of key trait innovations through allele combinations and recombination, it enriches the applications of pan-genome analysis and helps to reveal the origin of cultivated crops as well as the genes related to tuber formation. Pan-genome research can also be applied to genome sequencing of germplasm resources with significant differences among different ecogeographical types, enabling the mining of novel genes in species. This provides important information for studies on the supplementation of candidate genes, species diversity, adaptive evolution, origin history and invasiveness of alien species. For example, biogeographical analysis of soybean populations revealed that modern cultivated soybeans originated in northern China (Liu et al., 2020), while related studies on rice populations suggested that the origin of modern cultivated rice should include southern China (Huang et al., 2012). Constructing pan-genome that include materials from different domestication stages and geographical regions is of great significance for investigating crop evolution and domestication (Zuntini et al., 2024). Pangenomic analysis of apples showed that approximately 90% of genes in cultivated apples are derived from their wild ancestors *Malus sylvestris (L.) Mill*. and *Malus sieversii (Ledeb.) M. Roem* (Sun et al., 2020), confirming that apples were transmitted from China to Europe along the Silk Road and gradually domesticated into cultivated varieties. The re-domestication of wild allotetraploid rice, through the establishment of efficient transformation and genome editing systems based on high-quality reference genomes, has also provided new insights into the design of ideal crop species (Yu et al., 2021). In summary, pan-genome have expanded the depth and breadth of reference genomes, facilitating comprehensive analysis of crop evolution and domestication.

### 3.4 Mechanistic insights and comparative advantages of pan-genomic approaches

The accumulation of pan-genomic studies now allows for a critical, mechanistic comparison of its approaches against traditional linear reference frameworks. Graph-based pan-genomes demonstrate superior efficacy in scenarios characterized by high sequence diversity and complex structural variations. Linear references, by definition, collapse diversity into a single sequence, creating systematic reference bias. Read alignment to a linear reference often fails in these regions, leading to false negatives and mischaracterization of variation. A graph genome incorporates all major haplotypes as alternate paths, enabling accurate read mapping and variant calling across the entire spectrum of diversity (Garrison et al., 2018; Li et al., 2022).

PAV Hotspots: Genomic regions that are present in some individuals and absent in others are completely invisible to linear-reference-based analyses. Graph-based pan-genomes explicitly represent these PAVs, allowing for their discovery and association with phenotypes. For instance, in cucumber, graph-based assembly uncovered extensive SVs and PAVs linked to domestication traits that were missed in previous studies reliant on a single reference (Li et al., 2022). For breeding applications, understanding the phase of alleles—which combinations of variants are co-inherited—is crucial. Graph-based structures naturally facilitate more accurate haplotype resolution, which is essential for leveraging non-additive genetic effects like dominance and epistasis in genomic selection models.

Genome-Wide Association Studies (GWAS): Restricting GWAS to SNPs only can miss the genetic architecture of traits governed by gene gain/loss. Adding PAVs transforms GWAS in discovery of causal large-effect variants, many agronomically important traits are directly controlled by PAVs. For example, the presence of a specific gene allele, not a SNP within it, may confer resistance. Without testing for PAVs, the true causal variant remains hidden. In tomato, pangenomic GWAS identified PAVs associated with fruit flavor and size that were undetectable using SNP-based approaches alone (Gao et al., 2019; Alonge et al., 2020). Genomic Prediction (GP): The goal of GP is to accurately predict the breeding value of an individual based on its genotype. Models using only SNPs capture a portion of the genetic variance, primarily additive effects.

Compared with the method that only uses SNPs, the approach of including PAVs has completely transformed the downstream analysis. The specific analysis is as follows: Firstly, PAV can be the underlying causal variant for which nearby SNPs are merely markers. By including the PAV itself in the association model, it can eliminate false-positive signals from linked SNPs, thereby clarifying the true genetic architecture and leading to more reliable marker development for breeding. Finally, PAVs frequently have large phenotypic effects, their inclusion in GP models can significantly increase predictive accuracy for traits where gene presence/absence is a key determinant, effectively capturing a portion of “missing heritability” that SNP-based models fail to account for.

In conclusion, the transition to graph-based pan-genomes is an incremental improvement that requires continuous improvement of sequencing technologies, costs, and time. It is most impactful in species or genomic regions with high structural diversity. The integration of PAVs into analytical frameworks moves the field from a focus on sequence variation within genes to the broader and more functionally impactful spectrum of gene content variation itself. This provides a more complete biological picture, leading to more powerful discovery and more accurate prediction in plant genetics and breeding.

## 4 Limitations and challenges

The assembly of complex plant genomes characterized by high heterozygosity, repetitive content, and polyploidy, along with pan-genome construction (Sun et al., 2025), remains a significant challenge, it is necessary to develop specialized typing tools for polyploids, such as tetraDecoder combined with long-read sequencing, while integrating asymmetric analysis of subgenomes. Constructing pan-genomes for polyploid species adds a layer of complexity: phasing (assigning sequences to their respective subgenomes) and homoeolog resolution (distinguishing between highly similar copies of genes from different subgenomes) (Li et al., 2020). Instead of generating one assembly, the goal is to produce phased, haplotype-resolved assemblies for each accession. Failure to phase results in a chimeric assembly where homoeologs are collapsed, creating artificial, dominant alleles and obscuring true SVs and their effects. Plants like potato, grape, or many fruit trees accumulate high levels of heterozygosity and a heavy load of somatic SVs. For these species, a haplotype-resolved approach is not just beneficial but necessary to capture the full genetic diversity and understand traits like disease resistance or fruit quality, which are often allele-specific (Vaughn et al., 2022). Furthermore, methodological differences lead to incomparable analysis results, pan-genome construction strategies have a significant impact on the sensitivity and specificity of variant detection (Benoit et al., 2025), therefore, establishing a universal genome storage standard, such as the rGFA format, combined with the SV integration process, like Jasmine, is of crucial importance. Additional challenges arise when extending single-species pan-genome frameworks to entire genera, where higher divergence rates and lower sequence alignment efficiency complicate comparative analyses. Besides, during assembly, highly identical sequences from different genomic locations (e.g., recent segmental duplications, long terminal repeats of LTR retrotransposons) can be collapsed into a single consensus sequence. This collapse creates false absence calls in other accessions and artificial false, so SV calls should be filtered based on read support, genotype quality, and their location relative to annotated repeats, and combining long reads for continuity with short reads or Hi-C data for scaffolding and error correction (Zhu et al., 2025). Finally, the compatibility of cross-taxonomic pan-genome construction is also an issue that needs to be addressed urgently (Liang et al., 2025), it requires the use of Artificial intelligence (AI) models to predict the interactions of paralogous genes.

While plant pan-genome constitute invaluable resources for understanding genomic diversity and advancing plants genetics and breeding, this field faces critical challenges related to methodological standardization. Addressing these limitations is essential to ensure the reliability and robustness of pan-genomic analyses.

## 5 Future perspectives: advancing plant pan-genomics through integrated technologies

Plant pan-genomics has revolutionized our understanding of genomic diversity, yet its full potential remains untapped. This chapter outlines transformative research directions and methodological innovations poised to redefine crop improvement paradigms.

### 5.1 Graph-based and super-pan-genome: architecting the next-generation references

Current linear reference genomes fail to capture species-wide SVs. Graph-based pan-genome—representing sequences as nodes and connections as edges—enable precise haplotype resolution and complex SV mapping (Mishra et al., 2024). Tools like minigraph and PGGB now integrate phased assembly for polyploids. Super-pan-genome expansion: Genus-level graphs identify cross-species compensatory mutations, also facilitate the analysis of the time of species differentiation and evolutionary events, and can provide new resources and ideas for plant evolution research (He et al., 2025; Li et al., 2025b).

### 5.2 AI-driven functional prediction and breeding acceleration

Machine learning models now decode non-additive genetic effects hidden in pan-genomic SVs: Deep learning for variant effect prediction: Transformer models use epigenetic fingerprints to prioritize functional SVs (Li et al., 2025b). AI-driven phenomics shows particular promise for future breeding programs, facilitating the modernization of agriculture. Machine learning approaches that incorporate non-additive effects into GS will gain enhanced predictive power when coupled with pan-genome representing complete genomic repertoires, substantially accelerating plant breeding.

### 5.3 Multi-omics integration for closed-loop validation

Linking pan-genomic variants to phenotypic outcomes requires systematic validation. Future efforts must prioritize single-cell omics to resolve spatial regulation, such as from PAV to transcriptome to phenotype, and to strengthen the integration of wild relatives. Next-generation pan-genome-enabled tools for GWAS and QTL mapping will leverage multi-scale, high-density genetic variations to propel plant comparative genomics, functional genomics, and molecular breeding. Peanut pan-genome coupled with scRNA-seq uncovered *AhARF2–2* expression mosaicism in developing seeds, explaining size variation (Zhao et al., 2025).

The convergence of graph-based references, AI-driven prediction, and multi-omics validation will transition pan-genomics from descriptive tools to predictive breeding engines. Immediate priorities include: Deploying super-pan-genome for major crops; Establishing AI-optimized editing vectors for complex trait stacking, automated high-throughput phenotyping platforms will further empower molecular breeding, while larger-scale datasets and enriched data structures will surpass linear references in enhancing variant discovery and utilization.; Developing field-applicable PAV diagnostic kits. As global initiatives like CropPan unify data standards, pan-genomics will catalyze a 50% reduction in varietal development cycles, ushering in an era of precision-designed crops resilient to climate disruptions.
